# Search for inherited susceptibility to radiation-associated meningioma by genomewide SNP linkage disequilibrium mapping

**DOI:** 10.1038/bjc.2011.61

**Published:** 2011-03-01

**Authors:** F J Hosking, D Feldman, R Bruchim, B Olver, A Lloyd, J Vijayakrishnan, P Flint-Richter, P Broderick, R S Houlston, S Sadetzki

**Affiliations:** 1 Section of Cancer Genetics, Institute of Cancer Research, Sutton, Surrey, UK; 2 Cancer & Radiation Epidemiology Unit, Gertner Institute, Chaim Sheba Medical Centre, Tel Hashomer, Israel; 3 Sackler School of Medicine, Tel Aviv University, Tel Aviv, Israel

**Keywords:** ionising radiation, meningioma, genetic, sensitivity

## Abstract

**Background:**

Exposure to ionising radiation is a well-established risk factor for multiple types of tumours, including malignant brain tumours. In the 1950s, radiotherapy was used to treat Tinea Capitis (TC) in thousands of children, mostly of North-African and Middle Eastern origin, during the mass migration to Israel. The over-representation of radiation-associated meningioma (RAM) and other cancers in specific families provide support for inherited genetic susceptibility to radiation-induced cancer.

**Methods:**

To test this hypothesis, we genotyped 15 families segregating RAM using high-density single-nucleotide polymorphism (SNP) arrays. Using the family-based association test (FBAT) programme, we tested each polymorphism and haplotype for an association with RAM.

**Results:**

The strongest haplotype associations were attained at 18q21.1 (*P*=7.5 × 10^−5^), 18q21.31 (*P*=2.8 × 10^−5^) and 10q21.3 (*P*=1.6 × 10^−4^). Although associations were not formally statistically significant after adjustment for multiple testing, the 18q21.1 and 10q21.3 associations provide support for a variation in *PIAS2, KATNAL2, TCEB3C, TCEB3CL* and *CTNNA3* genes as risk factors for RAM.

**Conclusion:**

These findings suggest that any underlying genetic susceptibility to RAM is likely to be mediated through the co-inheritance of multiple risk alleles rather than a single major gene locus determining radiosensitivity.

A number of rare inherited cancer syndromes are typified by radiosensitivity, such as Gorlin and Li-Fraumeni syndromes (Gatti, 2001). Collectively, these diseases are, however, rare. Evidence that inherited sensitivity to radiation may have a more general genetic basis is provided by the observation that cancer patients and some of their first-degree relatives exhibit increased *in vitro* radiosensitivity compared with healthy controls (Roberts *et al*, 1999; Burrill *et al*, 2000).

Ionising radiation is the only environmental factor that has been shown unequivocally to be a causative factor for meningioma development (Sadetzki *et al*, 2005a; Bondy *et al*, 2008). During the mass migration to Israel in the 1950s, the Israeli authorities undertook a wide-scale campaign to eradicate Tinea Capitis (TC). The treatment included radiotherapy to the head area and was administered to children with TC in Israel and abroad, mainly in North-African and Middle Eastern countries, who were planning to immigrate to Israel. The therapeutic procedure followed the Adamson-Kienbock technique. The hair had been shaved and any remaining hair was removed through a waxing process. Subsequently, the scalp area was divided into five fields, each being treated on one of five consecutive days. The irradiation was done with a 75–100 kV superficial therapy X-ray machine. The children were exposed to 3.5–4.0 Gy for each field, at a focus skin distance of 25–30 cm. Most individuals received one course of radiation, but ∼9% of the patients received ⩾2 treatments (Werner *et al*, 1968).

A subgroup of children who were treated in Israel, including a group of 10 842 irradiated individuals with two matched nonexposed population and sibling groups (referred henceforth as the TC cohort), has been systematically followed for over 50 years for radiation sequelae. Radiation dosimetry was done for this cohort in the late 1960s using one of the original X-ray machines and a head phantom. These studies estimated the average dose to the brain at 1.5 Gy (s.d. 0.52, range 1.0–6.0 Gy). Doses were also calculated for different areas of the brain with the lowest average dose being for the back and front of the lower plane (mean 1.1 Gy, s.d. 0.37, range 0.71–4.30), whereas the highest dose was for the front of the upper plane (mean 1.8 Gy, s.d. 0.61, range 1.17–7.11) (Ron *et al*, 1988).

Although affecting <1% of the TC cohort, a marked increase in the risk of meningioma (ERR/Gy 4.63; 95% CI: 2.4–9.1) is one of the most prominent observations seen among the exposed individuals (Sadetzki *et al*, 2005a).

On the basis of the above-mentioned results, a law was established in Israel in 1994, for the purpose of compensating irradiated individuals who had developed specific diseases that were proven to be causally associated with the irradiation given as treatment for TC. The irradiated and nonirradiated cases and controls from the TC cohort, as well as irradiated cases who claimed compensation within the framework of the law, constitute the study population for nested case–control studies designed to assess interaction between ionising radiation and other environmental and genetic risk factors in the development of cancer (Sadetzki *et al*, 2005b; Flint-Richter and Sadetzki, 2007). These studies have demonstrated an over-representation of radiation-associated meningioma (RAM) and other radiation-associated cancers in specific families. This finding indicates that the occurrence of the tumour following the exposure is not a random event, and provides support for the hypothesis of inherited genetic susceptibility to radiation-induced cancers (Flint-Richter and Sadetzki, 2007).

The TC cohort is derived from a population that is characterised by high levels of linkage disequilibrium (LD). This allelic architecture affords enhanced power to localise and identify disease-causing alleles through association-based analyses especially if a restricted gene set underscores inherited susceptibility to RAM. In this study we report a search for RAM susceptibility alleles in TC families through an LD association-based analysis of genomewide single-nucleotide polymorphism (SNP) genotypes.

## Subjects and Methods

### Subjects

Our search for alleles predisposing to RAM was based on families ascertained through the TC studies. In total, 15 families in whom ⩾2 cases of RAM have been diagnosed among first-degree relatives were identified (Table 1). Of these families, 14 were identified from a larger epidemiological, genetic case–control study that included 160 RAM participants of whom 17 have reported on at least one sibling who was diagnosed with meningioma. However, out of these families, only 14 agreed to participate in the current study. More details on the methodology of this study have been previously published (Sadetzki *et al*, 2005b; Flint-Richter and Sadetzki, 2007). One additional family was recruited from the claim files, resulting in a total of 15 families. The target study population included 120 individuals (40 RAM, 14 healthy irradiated, 49 healthy nonirradiated, 9 irradiated with other cancer and 8 nonirradiated with other cancer); the number of siblings in each family ranged from 5 to 12. The age at diagnosis for the RAM patients ranged from 35 to 69 years (mean 48.7±9.2). Validation for irradiation status and for tumour pathology was performed for all of these family members, using medical records for pathology verification and a set of criteria that were used in previous studies (Sadetzki *et al*, 2005b) for irradiation verification.

Biological specimens were collected from 71 individuals; however, the final genetic analysis was based on 65 samples because only DNA extracted from peripheral blood (*n*=66, 27 RAM) was used and 1 DNA was excluded because of having an overall call rate of <95% (Table 1).

### Ethics

Collection of blood samples and clinicopathological information from subjects was undertaken with informed consent and relevant ethical review board approval in accordance with the tenets of the Declaration of Helsinki.

### Genotyping

DNA was extracted from EDTA-venous blood samples using conventional methods and quantified using PicoGreen (Invitrogen, Carlsbad, CA, USA). Genotyping was conducted using Illumina 610Quad arrays according to the manufacturer's protocols (Illumina San Diego, CA, USA). To ensure quality of genotyping, a series of duplicate samples was genotyped, resulting in 99.99% concordant calls. We excluded SNPs from analysis if they failed one or more of the following thresholds: GenCall scores <0.25; overall call rates <95% minor allele frequency (MAF) ⩽0.01; outlying in terms of signal intensity or X : Y ratio; discordance between duplicate samples; and, for SNPs with evidence of association, and poor clustering on inspection of X : Y plots.

### Statistical analyses

The primary analysis was for association of individual SNPs with the binary trait of RAM, using the family-based association test (FBAT) programme (Horvath *et al*, 2001), and for haplotypes, using the haplotype extension (HBAT) of the FBAT programme (Horvath *et al*, 2004). FBAT is a generalised version of the classical transmission–disequilibrium test, which can be applied to any type of nuclear family (Laird *et al*, 2000), thus avoiding the issue of population admixture that is a commonly encountered in case–control study designs. In the FBAT programme, the additive model was used.

Haplotype analyses were performed using sliding window sizes of 12 contiguous markers. Haplotype frequencies for each individual were estimated using an expectation-maximisation (EM) algorithm. The minimum haplotype frequency was set at 0.01, and haplotypes with frequencies below this threshold were combined into a single group.

Because of the large number of multiple tests performed, we used the Benjamini and Hochberg correction (Benjamini *et al*, 2001) for multiple testing, which is a method for controlling the false discovery rate, to adjust the haplotypic *P-*values. This correction consists of ranking all the *P-*values, from smallest to largest, and adjusting each by multiplying by the total number of tests and dividing by the rank of that *P-*value. All test statistics with rank less than the test statistic with the largest rank for which the corrected value is less than the desired error rate (e.g., 0.05) are significant.

### Mutational analysis

A search for mutations in the coding regions and splice sites of all isoforms of *CTNNA3* and *LRRTM3,* as annotated by GRChB37, was performed by sequencing amplified PCR fragments using BigDye Terminator chemistry implemented on an ABI 3730xl sequencer (Applied Biosystems, Carlsbad, CA, USA). PCR primers were designed using Primer 3 software and are given in Supplementary Table 1. Sequence traces were aligned and compared with the gene consensus sequence using Mutation Surveyor (Version 3.2; SoftGenetics, State College, PA, USA). Two *in silico* algorithms, PolyPhen (http://genetics.bwh.harvard.edu/pph/) and SIFT (http://sift.jcvi.org), were used to predict the putative impact of missense variants on protein function. Scores were classified as tolerated, borderline or deleterious according to the proposed criteria.

## Results

**Figure 1:**
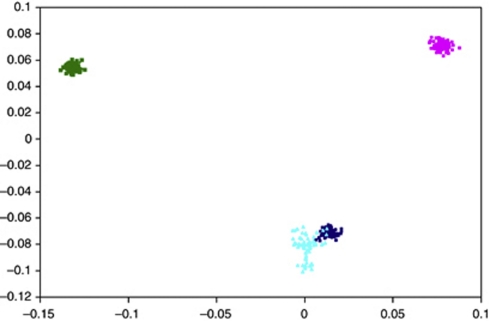
Principal component analysis of SNP genotypes showing the extent of ethnic variability in the TC cohort. The first two principal components of the analysis were plotted. HapMap CEU (Caucasian) individuals are denoted by grey triangles, CHB (Chinese Han Beijing)+JPT (Japanese in Tokyo) by grey diamonds, YRI (Yoruba) by grey squares and TC cohort individuals are plotted in black.

Illumina 610Quad SNP genotypes were obtained for all 66 samples genotyped. Before conducting association-based analyses, we subjected the SNP data set to rigorous quality control in terms of excluding samples and SNPs with poor call rates. As mentioned previously, one sample was excluded because of having a call rate of <95% the remaining samples had average call rates across all SNPs of >99%. Thus, the final analysis was based on 65 samples. Following this, we critically evaluated the data set for ancestral differences by principal component analysis (Figure 1). Although minor differences were apparent, all individuals genotyped were relatively ancestrally comparable. Thus, without introducing significant systematic bias we considered the data set to be uniform to maximise power to detect important associations under the assumption of homogeneity and an ancestral risk haplotype for RAM. In all analyses we treated individuals with RAM as affected and all other family members as of unknown phenotype.

The median distance between the 575 272 autosomal SNPs in the Illumina 610Quad arrays was ∼2.7 Kb and ∼88% of the genome was within 10 Kb of a SNP marker. In this study, the heterozygosity of markers was ∼94%, hence almost as many SNPs present on that array are heterozygous in this Jewish population as in the general Caucasian population.

We systematically interrogated haplotypes defined by a varying number of SNPs. Haplotypes defined by >12 SNPs proved too computationally intensive to recover on a genomewide basis. We therefore restricted our search for disease-associated risk locus on the basis of 12 SNP haplotypes.

**Figure 2:**
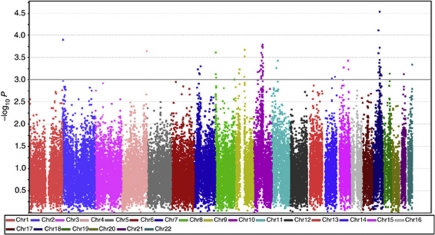
Manhattan plot of genomewide haplotype test *P-*values for the association between haplotypes and RAM. The –log_10_ *P-*values (*y* axis) are presented at their chromosomal positions (*x* axis).

This analysis provided results on the association between RAM and 41 414 haplotype tests across the genome (Figure 2). In all, 66 haplotype tests provided evidence for an association between genotype and RAM at *P*<0.001 (Table 2) including multiple haplotypes on chromosomes 18 and 10. The strongest associations were shown at 18q21.1 (*P*=7.5 × 10^−5^), 18q21.32 (*P*=2.8 × 10^−5^) and 10q21.3 (*P*=1.6 × 10^−4^).

A number of genes map to the 18q21.1 region of association including *PIAS2, KATNAL2, TCEB3CL, TCEB3C, TCEB3B* and *DKfZp667C165,* whereas the 18q21.31 region is bereft of genes. In contrast to the other associations, the 10q21.3 signal was characterised by a large number of neighbouring haplotype associations; eight providing evidence for an association at *P*<0.001. These haplotypes all mapped within a 2 Mb region of 10q21.3 and all annotate the *catenin* (*cadherin-associated protein*), *α*-*3* (*CTNNA3*) gene. Among the top 66 associations, we identified only two other genes that were annotated by multiple haplotype tests showing evidence for an association at *P*<0.001. Specifically, *TMC1* on 9p21.13 and *PCDH15* on 10q21.1 were captured two and three times, respectively, by haplotype associations (Table 2).

**Figure 3:**
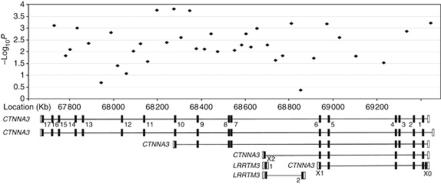
Haplotype –log_10_ *P*-values for the 10q21.3 region. Beneath the plot are the five isoforms of *CTNNA3* and two of *LRRTM3* with exons shown as black blocks and UTRs as empty blocks, annotated as per Supplementary Table 2.

The CTNNA3 is part of the Wnt signalling pathway and, although speculative, *CTNNA3* represents an attractive basis for susceptibility given the role of dysfunctional Wnt signalling in radiosensitivity. In view of this, we explored the possibility that a common or restricted set of coding sequence changes in *CTNNA3* might underscore the 10q21.3 association. For completeness we also screened the *leucine rich repeat transmembrane neuronal 3* (*LRRTM3*) gene that maps internally within *CTNNA3* (Figure 3).

Nine sequence changes within coding sequence were identified in the same 65 individuals whose DNA passed QC in the genomewide stage. These included five polymorphic variants documented in dbSNP (four in *CTNNA3* and one in *LRRTM3*) and four novel changes (three and one in *CTNNA3* and *LRRTM3*, respectively). Seven of the variants identified were missense changes, six in *CTNNA3* and one in *LRRTM3*. None of the missense changes identified were confined to individuals with a RAM phenotype (Supplementary Table 2). Furthermore, none of the sequence changes were predicted to impact on the functionality of the expressed protein.

## Discussion

The TC cohort is unique, and has allowed us to recently assess the impact of environmental and inherited risk factors on tumour development in those exposed to ionising radiation (Bondy *et al*, 2008). In contrast to the rarity of familial meningioma in the general population, 17 families within the TC study had two or more members affected with RAM, equating to sibling relative risk of ∼20-fold. As the doses of therapeutic radiation administered to individuals within the TC cohort were similar (interquartile range (25–75%) 127–153 cGy), it has raised the possibility that the impact of ionising radiation on cancer risk is in part a consequence of genetic susceptibility conferred by low penetrance genes.

To provide evidence for this hypothesis and identify a RAM-associated disease locus, we have conducted an associated analysis using high-density SNP genotyping. The SNP LD mapping strategy employed in this study has relied on the comparatively large regions of LD that encompass founder mutations segregating in the Jewish population. This simple study design strategy and using DNAs from a small number of people has previously been successfully used to localise a susceptibility gene for Bloom's syndrome (Mitra *et al*, 2004).

Predicated on the assumption of inherited predisposition, our study provides insight into the possible architecture of genetic susceptibility to RAM. Over a range of gene frequencies of 0.001 to 0.05, and stipulating a false positive rate of 0.0001, our study had high power (>70%) to identify a disease-associated haplotype contributing >30% of the excess risk assuming a simple genetic model of familial aggregation. Although assuming an effect size of 30% is high for many complex traits as previously articulated, an assumption on which our study was predicated is that RAM is primarily a consequence of major gene susceptibility and because of the restricted ethnicity allelic heterogeneity is limited.

An alternative model of RAM is that this phenotype is a consequence of a complex-polygenic basis. Failure to unambiguously identify a single locus is thus entirely compatible with the latter model of disease susceptibility, whereby disease risk is mediated by alleles conferring more modest effects, possibly through the consequence of the co-inheritance of multiple low-risk variants. Under this model, we would have had only very limited power to identify a disease-causing locus, stipulating a *P-*value of 1 × 10^−6^ to ensure genomewide significance. At the lower significance threshold, our analysis does provide some evidence to support the involvement of a number of genes in the aetiology of RAM; specifically, the gene encoding *CTNNA3* that is captured by the 10q21.3 haplotypes. This gene is of specific interest as it is part of the Wnt signalling pathway, which has been related to cancer development and neurodegeneration. Several components of the Wnt pathway have been implicated in carcinogenesis and are best known to be involved in colorectal, lung, prostate, breast and skin cancers (Behrens *et al*, 2009; Lai *et al*, 2009). Moreover the *CTNNA3* gene contains a fragile site of potential interest in terms of genomic instability as there is evidence suggesting that it may function as a tumour suppressor (Smith *et al*, 2006).

Although there is evidence for inherited susceptibility to radiosensitivity outside the context of a restricted set of syndromes, it is primarily derived from *in vitro* data. However, the phenotype radiosensitivity is relatively prosaic, and establishing a relationship between genotype and sensitivity is inherently problematic as multiple clinical end points can be considered, many of which are ill defined. To obviate this, we have made use of a unique cohort and have sought to establish a relationship between constitutional genotype and cancer risk.

Failure in our study to unambiguously identify a single high-risk locus provides evidence for a model of inherited susceptibility to radiosensitivity based on the co-inheritance of multiple low-risk variants. Although individually such loci only confer small effects, it is likely that they act multiplicatively, exerting relatively profound effects in a small proportion of the population.

## Figures and Tables

**Table 1:** Description of RAM families having two or more members with meningioma among siblings

								**Final population for genetic analysis**				
									**RAM**		**Non-RAM siblings**	
**Family ID**	**Ethnic origin**	**No. of siblings with RAM**	**Other cancers in irradiated siblings**	**Other cancers in nonirradiated siblings**	**No. of healthy irradiated siblings**	**No. of healthy nonirradiated siblings**	**Total no. of siblings**	**No. of blood samples** ^*^	**No. of siblings with RAM**	**Ages at diagnosis**	**No. of nonirradiated siblings**	**No. of irradiated siblings**
1	Libya	3	0	0	1	4	8	3	1	69	1	1
2	Morocco	4	0	0	1	2	7	7	4	32, 37, 52, 54	2	1
3	Morocco	2	0	Lymphoma	0	5	8	5	2	35, 45	3	0
4	Morocco	3	Breast, leukaemia	0	2	3	10	5	1	49	2	2
5	Morocco	2	Leukaemia	Mole	2	3	9	5	2	39, 42	2	1
6	Yemen	5	0	0	0	2	7	3	2	53, 55	1	0
7	Yemen	2	0	Lung	0	5	8	6	1	69	5	0
8	Morocco	2	Colon	0	1	3	7	3	2	40, 52	1	0
9	Morocco	3	Leukaemia	Liver, leukaemia	1	4	11	1	1	54	0	0
10	Morocco	2	0	0	0	3	5	1	1	37	0	0
11	Morocco	3	0	0	0	5	8	8	3	45, 47, 51	5	0
12	Iraq	2	Breast, BCC	Colon, lung, lung	3	2	12	6	2	42, 49	2	2
13	Iran	2	0	0	1	2	5	3	1	52	2	0
14	Morocco	2	BCC	0	2	4	9	4	2	46, 57	1	1
15	Iran	3	0	Breast	0	2	6	5	2	56, 56	3	0
**Total**		**40**	**8**	**9**	**14**	**49**	**120**	**65**	**27**		**30**	**8**

Abbreviations: BCC=basal cell carcinoma; RAM=radiation-associated meningioma.

^*^One sample from family ID 4 was excluded because of call rate <95%.

**Table 2:** Details of haplotypes showing evidence of association with RAM at *P*<0.001

**Chr**	**Start SNP**	**End SNP**	**Location**	***P*-value**	**Enclosed gene(s)**
2p25.3	rs10174217	rs13431590	2,742 650–2 761 577	0.000317	
4q35.2	rs4253236	rs925453	187 148 071–87 179 210	0.00022	*KLKB1*
7p14.3	rs1544470	rs12700916	28 847 869–28 896 396	0.000569	*CREB5*
7p14.1	rs2280668	rs7777684	39 172 024–39 245 404	0.000705	*POU6F2*
7p14.1	rs6949528	rs12701960	42 261 852–42 314 044	0.000726	*GLI3*
7p12.3	rs1025521	rs17662528	48 492 470–48 508 408	0.000487	*ABCA13*
8p23.2^*^	rs2618841	rs10503179	2 151 954–2 304 545	0.000234	
8p23.2^*^	rs13259957	rs35909721	2 304 723–2 324 045	0.000731	
8p23.2	rs11136914	rs7831044	5 533 388–5 545 090	0.000876	
8q24.3	rs7822130	rs4076117	141 242 366–141 247 104	0.000964	*TRAPPC9*
9p21.1	rs10970796	rs10970826	32 128 828–32 176 938	0.000693	
9p13.3	rs1571401	rs2812357	34 572 815–34 655 436	0.000574	*CNTFR, C9orf23, DCTN3, ARID3C, SIGMAR1, GALT, IL11RA,*
9q21.12	rs10868893	rs2039646	73 419 560–73 452 088	0.000837	*TRPM3*
9q21.13^*^	rs7851040	rs17095	75 144 401–75 206 337	0.00029	*TMC1*
9q21.13^*^	rs7029452	rs17058062	75 207 329–75 262 770	0.000207	*TMC1*
10p11.22	rs703069	rs4747759	31 933 997–31 974 332	0.00066	
10q11.21	rs11238782	rs4948591	44 438 863–44 490 414	0.00063	
10q21.1	rs4935365	rs1343041	54 629 426–54 665 442	0.000566	
10q21.1	rs10824952	rs4144618	54 991 869–55 046 704	0.000466	
10q21.1	rs1930145	rs11004362	56 286 068–56 302 374	0.000813	*PCDH15*
10q21.1^*^	rs2488843	rs2488827	57 062 207–57 134 988	0.00024	*PCDH15*
10q21.1^*^	rs1777675	rs1334526	57 139 743–57 183 477	0.000921	*PCDH15*
10q21.1	rs1769039	rs714113	59 953 829–60 061 939	0.000361	*IPMK, CISD1, ZCD1*
10q21.2	rs10995111	rs2087625	64 060 268–64 117 756	0.000273	*ZNF365*
10q21.3	rs224285	rs10509173	64 584 810–64 611 889	0.000479	
10q21.3	rs2619601	rs12357769	65 461 640–65 520 368	0.000171	
10q21.3^*^	rs7091769	rs7898508	66 529 552–66 585 513	0.000306	
10q21.3^*^	rs16920432	rs2932842	66 586 374–66 662 925	0.000604	
10q21.3	rs17205485	rs953458	67 057 880–67 082 879	0.000712	
10q21.3	rs1941993	rs4746538	67 687 416–67 751 473	0.000785	*CTNNA3*
10q21.3	rs1903863	rs10822705	67 803 410–67 842 734	0.000989	*CTNNA3*
10q21.3	rs2456750	rs1911323	68 164 444–68 217 760	0.000176	*CTNNA3*
10q21.3	rs1911343	rs997225	68 257 630–68 282 970	0.000156	*CTNNA3*
10q21.3	rs10997235	rs10822851	68 324 435–68 361 408	0.000186	*CTNNA3*
10q21.3	rs2394319	rs2394323	68 794 756–68 827 014	0.000645	*CTNNA3, LRRTM3*
10q21.3	rs7091927	rs10509284	68 949 597–69 004 536	0.000662	*CTNNA3*
10q21.3	rs16924708	rs932656	69 380 156–69 530 678	0.000628	*CTNNA3*
10q21.3	rs4558056	rs3740593	70 401 976–70 501 910	0.000514	*TET1, CCAR1*
10q22.1	rs1163179	rs1498325	70 610 209–70 636 836	0.000514	*STOX1*
11p13	rs11032695	rs2284369	34 447 586–34 468 936	0.000531	*CAT*
11p11.2	rs4755854	rs7123370	44 649 231–44 694 382	0.000362	
14q24.2	rs193444	rs17178387	71 817 538–71 997 723	0.00092	*SIPA1L1*
14q32.12	rs4900155	rs4905002	93 316 522–93 350 376	0.000834	
15q21.1	rs999128	rs1865649	47 895 214–47 939 505	0.000982	*SEMA6D*
15q21.3	rs958760	rs573740	54 636 344–54 683 431	0.000527	*UNC13C, HO74*
15q25.3	rs2346715	rs720736	87 081 060–87 136 204	0.000365	*AGBL1*
15q26.1	rs293380	rs1125105	89 645 230–89 689 964	0.000574	*ABHD2*
18q21.1	rs11662257	rs1878059	44 204 073–44 234 546	0.000673	
18q21.1^*^	rs4121690	rs2032215	44 344 852–44 439 011	0.000245	*PIAS2*
18q21.1^*^	rs12454431	rs2576042	44 451 644–44 577 461	0.000075	*PIAS2,KATNAL2,TCEB3CL/C/B, DKfZp667C165*
18q21.2^**^	rs1364417	rs2286812	53 693 949–53 717 464	0.000405	
18q21.2^**^	rs17733784	rs764699	53 719 925–53 745 508	0.000491	
18q21.31	rs967044	rs727453	53 958 845–54 045 333	0.000351	
18q21.31	rs1942336	rs4077610	54 778 102–54 843 053	0.000185	*FAM44C, BOD1P*
18q21.31	rs644016	rs12967876	55 164 547–55 191 037	0.000627	
18q21.31^*^	rs2663862	rs8094024	55 479 671–55 516 213	0.000779	
18q21.31^*^	rs12966493	rs12953872	55 517 038–55 580 836	0.000028	
18q21.32	rs4643439	rs4261640	56 694 779–56 732 513	0.000921	
18q21.32^*^	rs2271731	rs11660643	56 826 077–56 852 972	0.00056	
18q21.32^*^	rs7228554	rs9319943	56 858 758–56 879 827	0.000676	
18q21.33	rs500424	rs495005	60 540 860–60 597 508	0.000727	*PHLPP*
18q21.33	rs1589593	rs8088231	61 398 078–61 457 669	0.000515	*SERPINB7*
18q22.3	rs7227719	rs8095198	69 119 371–69 160 768	0.000787	
19q13.32	rs448784	rs8102349	48 764 721–48 832 554	0.000715	*ZNF114, CCDC114, EMP3, DKFZp434D2472*
21q22.11	rs13051785	rs2834315	35 323 286–35.355 410	0.000731	*CR626360*
22q13.33	rs7290681	rs138220	50 492 235–50 550 808	0.000443	*TTL8, MLC1, MOV10L1*

Abbreviations: SNP=single-nucleotide polymorphism; RAM=radiation-associated meningioma.

Chromosomal coordinates derived from the Genome Reference Consortium GRChB37.

^*^, ^**^Denote haplotypes of length 12 that are consecutive.
